# Green Public Procurement at a Regional Level. Case Study: The Valencia Region of Spain

**DOI:** 10.3390/ijerph16162936

**Published:** 2019-08-15

**Authors:** Jose Luis Fuentes-Bargues, Pablo Sebastian Ferrer-Gisbert, Mª Carmen González-Cruz, María Jose Bastante-Ceca

**Affiliations:** GIDDP, Departamento de Proyectos de Ingeniería, Universitat Politècnica de València, Camino de Vera s/n, 46022 Valencia, Spain

**Keywords:** green public procurement, tendering, environmental criteria, public works, Valencia region

## Abstract

Research on current practices and the state of green public procurement enables the identification of areas that can be improved, as well as opportunities to improve the tendering procedures from an environmental point of view. To understand the behaviour of local, provincial, and regional administrations concerning green public procurement, a case study on the Valencia region of Spain is made. The Valencian region is one of the most important communities in terms of population, number of contracting authorities, and weight in the Spanish Gross Domestic Product. In this study, a total of 967 procedures were analysed from calls for tenders made by municipal, provincial, and regional administrations in2016 and 2017.The results of this study show that the use of environmental criteria is 19.7% and the average weight is 4.1 out of 100. The civil engineering subsector, more than the building subsector, employs environmental criteria, particularly in projects tendered by regional administrations, whereas for projects with large budgets the level of use is similar for both subsectors. It is necessary to encourage plans to improve Green Public Procurement (GPP) practices in the Valencian administrations, especially those with a local scope such as municipalities.

## 1. Introduction

Public authorities are one of the main consumers of products, services, and works, and can play a crucial role in making consumption more sustainable [1,2]. Between 10% and 15% of the gross domestic product (GDP) of developed countries is allocated to public procurement [3,4,5,6,7], often linked to investments with high environmental impacts [8].

The United Nations defined sustainable public procurement (SPP) as a ‘procurement wherein an organisation uses its buying power to signal preferences to the market with its choice of goods and services that meet sustainable criteria’ [9]. Environmental aspects have been gaining relevance in the public policies of the SPP [10], and giving rise to the concept of green public procurement (GPP). The European Commission defined it as a ‘process whereby public authorities seek to produce goods, services, and works with a reduced environmental impact through their life cycle when compared to goods, services, and works with the primary function that would otherwise be procured’ [11].

Administrations have the potential to guide production and consumption trends by encouraging demand for environmentally friendly products and services [12,13,14,15]. For this reason, interest in GPP has increased significantly in recent years [16]. And within the study of the GPP it is interesting to know the behavior of local and regional administrations, as they are very important in the volume of global recruitment and their projects have a more direct impact on the population.

The construction sector is one of the most important sectors in terms of the total amount spent and number of employees. Although it represents slightly less than 10% of the European Union’s GDP, and employs 7% of the workforce [17], it implies a considerable impact on the environment, since it consumes more than a third of the world’s resources [18] and a similar percentage of the final energy in the OECD (Organization for Economic Co-operation and Development) [10].

An analysis of the adoption of GPP can be carried out from three approaches: technical specifications; award criteria; and conditions of execution of the contract [10,19]. Of these, the second is the most frequently used method, as it is difficult to discern whether sustainability is a minimum condition (normative), or a desire of the project promoter. The conditions of execution are usually very general and common to many [19,20,21].

The main objective of this research is to analyse the greenness of bidding documents in public tendering processes of public works in the Valencia region of Spain. The results will contribute to knowledge about GPP practices and help technical staff understand the current situation when encouraging the use of environmental criteria.

The paper is structured as follows. Section 1 presents the introduction. Section 2 describes previous research on GPP and the regulatory framework in the European Union and Spain, and defines the Valencia regional structure, and Section 3 develops the method. Section 4 shows the results, and Section 5 presents the discussion, and the results of this study are compared with results from other GPP studies. Finally, Section 6 presents the conclusions.

## 2. Background and Previous Findings

The current EU regulatory framework for public procurement is Directive 2014/24/EU [22] (which repealed Directive 2004/18/EC) [23]. Both standards refer to the environmental characteristics to be considered. In Spain, Directive 2004/18/EC was transposed into Act30/2007 [24], which became a consolidated text in Royal Decree 3/2011 [25]. Whereas Directive 2014/24/EU was transposed into Act9/2017 [26], which entered into force in March 2018. According to this legislation, contractual requirements must consider environmental criteria such as reduced emissions, noise, reduced consumption of resources, etc. Although some authors [27] warn about the difficulty of monitoring environmental conditions during the execution of a project, the adopted criteria should preferably be measurable, since one of the main difficulties in the application of the GPP is the vagueness and lack of clarity in its definition [2,8,28].

In the last decade, GPP has become one of the fundamental pillars of environmental and procurement policies in the European Union [29,30,31] and worldwide (USA [32], China [28], Hong Kong [33]), including developing countries such as Malaysia [34] and Vietnam [35].

One of the most important initiatives at a European level is Procura+, which started in 2000 and consists of a network of European public authorities and regions that connect, exchange, and act on sustainable and innovation procurement (more information at www.procuraplus.org). There are currently more than 40 European public authorities that communicate on sustainable and innovation procurement, sharing experiences and knowledge. The Procura+ initiative published the third edition of the ‘Procura+ Manual’ [36] in 2016, which follows the transposition of the 2014 Procurement Directive, to facilitate the strategic use of public contracts for broader societal goals.

The Marrakech process is another important initiative related to GPP practices. It was initiated by the United Nations in 2003 as a part of the Johannesburg Plan of Implementation, which recognised sustainable consumption and production as an overarching objective, and an essential requirement for sustainable development [37].

In 2006, Bouwer etal. [38] analysed 865 responses to questionnaires and processed 1000 tender documents in EU countries. They identified two groups related to the use of GPP: the ‘green 7’ (Austria, Denmark, Finland, Germany, United Kingdom, Netherlands and Sweden); and the ‘other 18’. In agreement with other authors [19,21,29], they also found that public authority staff exaggerated the implementation of GPP.

A similar study was carried out in 2012 [39]. This was based on a questionnaire to collect information from contracting authorities on the latest contracts signed (2009–2010) for 10 groups of procurement products/services. A total of 18,517 questionnaires were sent out and a total of 856 questionnaires were collected—a 4.6% response rate. As an average percentage for all product groups and for the whole EU, 26% of the contracts were ‘green’ (i.e., they used all the basic environmental criteria indicated by the EU). This percentage increased to 55% if it was limited to contracts that used at least one of the basic environmental criteria.

With regard to the use of environmental criteria in the countries of the European Union, the results show Finland in first place, with a percentage of over 80%. In second place, the Netherlands, Hungary, Lithuania and Latvia, between 60% and 80%. Italy, Austria, Belgium and Romania were between 40% and 60%. Slovenia, Denmark, Sweden, Germany, Spain and the Czech Republic were between 20% and 40%, and finally, a group of 11 countries with less than 20% (Bulgaria, Cyprus, Poland, Greece, Slovakia, France, Estonia, Malta, United Kingdom, Ireland, and Portugal). Luxembourg did not provide sufficient responses for classification.

One of the most significant results is that countries belonging to the ‘Green 7’ group in the 2006 study—Germany, Great Britain, Sweden or Denmark—presented much worse values.

Between 2003 and 2005, Nissinen et al. [40] studied a sample of tenders in Denmark, Finland and Sweden. A total of 335 sheets were analysed, 155 in 2003, and 180 in 2005. An analysis of the content of the contract documents was carried out and all the environmental criteria in the documents was recorded. In Finland, 28% of procedures with environmental criteria were registered in 2003 and 57% in 2005; in Sweden, 60% in 2003 and 80% in 2005; and in Denmark 60% in both years.

The environmental aspects of products included in the public procurement process were classified according to the CPV (common procurement vocabulary) and three blocks were distinguished: high, medium, and low. In the so-called ‘high’ group, products were defined as those related to paper and pulp, printing materials, and articles for printing, computers, and computer equipment, etc. The ‘middle’ group included products related to agriculture, clothing, and textiles, furniture, construction, repair and maintenance of installations. The ‘low’ group mainly comprised of cultural and sporting products, postal services, engineering and architectural services, and water transport services.

The authors also noted the difference between ‘environmental criteria’ and ‘well-defined environmental criteria’, calling a ‘well-defined environmental criterion’ as one for which the purchasing authority has given the information on how a criterion must be fulfilled and verified.

Carlsson and Waara [29] studied green procurement in Sweden in 2004, analysing a sample of 558 public authorities. As a result, they identified several constraints on the implementation of GPP: The high cost of sometimes green products; the lack of administrative resources and adequate environmental expertise; and possible complaints from unsuccessful bidders about insufficiently well-defined criteria. In a similar way, but for municipalities in Tuscany (Italy), Testa et al. [2] concluded that it is necessary to develop successful strategies, well-trained staff, and to have guidelines and tools for GPP.

Parikka-Alhola et al. [41] examined the use of GPP in Finland, Sweden, and Denmark in 2005. They found that almost one-third of the tenders contained environmental criteria with an average weight of 3.3%.

Michelsen et al. [42] studied green procurement practices at local and regional levels in Norway in 2007. The results showed that GPP is much more established in large municipalities than in small ones, with more resources available to establish a purchasing department with greater knowledge and selection criteria. For the Norwegian ICT (information and communication technology) sector, Igarashi et al. [43], observed that environmental criteria were the third most frequent award criterion after price and quality, but with a lower weight than the others.

In an econometric analysis, Testa et al. [44] analysed the factors influencing the adoption of GPP practices on authorities from municipalities and provinces in three Italian regions (Lazio, Liguria, and Emilia Romagna). Data was collected from 156 public authorities by telephone interview using a questionnaire of 15 points distributed into sections regarding public authority, awareness on environmental issues at strategic level, and measurement of public ‘green initiatives’ towards citizens and markets. The authors also distinguished several stages of the tendering process where the environmental criteria can be included, and according to the answers of the interviewees, 54% of the public authorities stated that environmental criteria are included in the technical specifications, 35% in assessing the technical expertise of competitors, 23% in choosing the award criteria, and 10% in the execution of supply or service contract

In Spain, the Aalborg Commitments [45] were established together with the final declaration of the Fourth European Conference on Sustainable Cities and Towns (Aalborg+10). It outlined the challenges faced by European municipalities on their pathway towards sustainability, together with the commitment to sustainable purchasing and the active promotion of sustainable production and consumption, in particular, eco-labelled, organic, ethical, and fair-trade products. A ‘Green Public Procurement Plan for the General State Administration and its Public and Social Security Bodies’ was established in 2008 [46]. An integrated national waste plan was agreed in Spain for 2008–2015 [47].

### 2.1. Research on GPP Construction Sector

The construction sector is a significant user of natural resources and energy [17]. As a result, this sector has been largely responsible for environmental pollution and problems related to sustainability. Research and policy on GPP in the construction sector has not received as much attention as products and services [48,49], but interest has increased in recent years.

Värnas et al. [50] carried out a study on public procurement of construction works in Sweden based on a survey (they obtained 48 responses from 62 questionnaires sent) and subsequent interviews. Among the results they obtained, the most commonly used criterion was the possession of an environmental management system (EMS). Other criteria used were environmental knowledge of the organisation and management of environmental aspects, which are described in the environmental plan. In the building subsector, the type of machinery (air conditioning) and the energy that the building will use in its final activity have also been used as environmental criteria. The maximum weight of environmental criteria in these procedures was 10%.

In a study on GPP in Italy, Testa et al. [19] concluded that 19% of public procurement used environmental specifications among the award criteria, the most frequent being energy efficiency. However, when the analysis was restricted to the economically most advantageous tenders (EMAT), this percentage amounted to 87% with an average weight of 18%. The authors found no correlation between the size of the public authority and the greenness of the offers.

In a study on environmental criteria in Spanish public works, Fuentes-Bargues et al. [20] analysed a sample of 100 projects tendered between 2008 and 2011, and found that the use of environmental criteria was around 35% with an average weight of 5.7%. The most commonly used criterion was the environmental action plan (EAP), although it lacks a clearer and more complete definition of content. In 2017, Fuentes-Bargues et al. [51] analysed environmental criteria in the public works of Spanish universities over a sample of 316 projects tendered between 2016 and 2017. Some19.2% of the tender documents used environmental criteria and the weight was 6.5 points out of 100. The most used criteria were improvements in the energy efficiency of the equipment, installations, and buildings.

The use of environmental criteria for construction sector tenders in the last three studies is below the European average of 53% according to the study of Renda et al. in 2012 [39] regarding GPP in the EU27. This is by far the lowest figure of all the product groups identified—although only 3% of the contracts analysed in this study can be called completely ‘green’ because they use all the basic environmental criteria indicated by the EU. With regard to the basic environmental criteria for the construction sector, the results of the survey showed that waste management was specifiedin40% of the contracts analysed, energy efficiency in 32%, recyclable/reusable materials in31%, water-saving facilities in 19%, and technical experience in 17%.

In reference to the transcendence of EMS, there are contradictory opinions. Lam et al. [52] conducted a study in China that concluded that the possession of an EMS by a construction company was not related to different behaviour with respect to environmental specifications. However, Testa et al. [2] concluded that the possession of a certified EMS by Italian construction companies had an influence on the percentage of green tenders obtained. From a strictly legal point of view, in Spain, the possession of a certified EMS can only be used as a criterion for the technical solvency of the tenderer, and not as a criterion for the award of a contract [53].

The European Commission published in May 2019 a revised version of the ‘GPP Training Toolkit’ consisting of six independent modules and ten operational modules, with PowerPoint presentations and accompanying guidance. Among the operational modules, we can find two related to construction: Module 7.5: Office Building Design, Construction and Management; and Module 7.7: Road Design, Construction and Maintenance [54,55]. The objective of this toolkit is to support contracting authorities (public authorities) in how they handle green procurement. Two levels of ambition were defined for each set of criteria: core and comprehensive. The first level is designed to allow an easy application of GPP, focusing on the key areas of environmental performance of a product and aimed at keeping administrative costs for companies to a minimum. The second level considers more aspects of higher levels of environmental performance for use by authorities that want to go further in supporting environmental and innovation goals [56].

The basis for both levels is the life cycle assessment (LCA); the use of environmental product declarations (EPDs), levels of CO_2_emissions; the use of materials with recycled and reused content; and the requirement to reduce emissions from the transport of heavy materials. All these criteria are lines of research related to GPP in constant development. For example, Butt et al. [57] studied in 2015 the use of an LCA in road construction contracts. They concluded that the methodology was not yet integrated into normal practice and that the limits of LCAs depended on the hierarchy of the decision level and the stage of the planning process. Similar conclusions were obtained by Lenferink et al. [58] in an investigation of design, construction, financing, and maintenance (DBFM) contracts in Dutch infrastructure projects. Along the same lines, Vidal et al. [59] during the development of a method for the integration of GPP into the LCA and TOPSIS (technique for order of preference by similarity to ideal solution) pointed out that the use of LCA tools requires highly trained practitioners and that most practitioners do not currently have such staff or training.

Other tools such as carbon footprint (CF) analysis can also be used in the GPP, as in the case study carried out by Alvarez and Rubio [60] on the waterfront of a 30-km stretch of the Manzanares River in Madrid (Spain).

It can be concluded from an analysis of these studies that it is necessary to improve the construction sector’s capacities to plan and implement GPP, so that the sector is prepared for anticipated opportunities and challenges.

One of these challenges (and according to the European Directives on public procurement) is identification of the most economically advantageous tender through a cost-effectiveness approach, such as Life Cycle Costing (LCC). The LCC allows to determine the total cost of an item from its conception and fabrication to the end of its useful life. Currently, successful LCC adoption is limited to a few cases, but its development and application is associated with contraction authorities with major experience in GPP [61].

### 2.2. Structure of the Valencia Region

**Figure 1:**
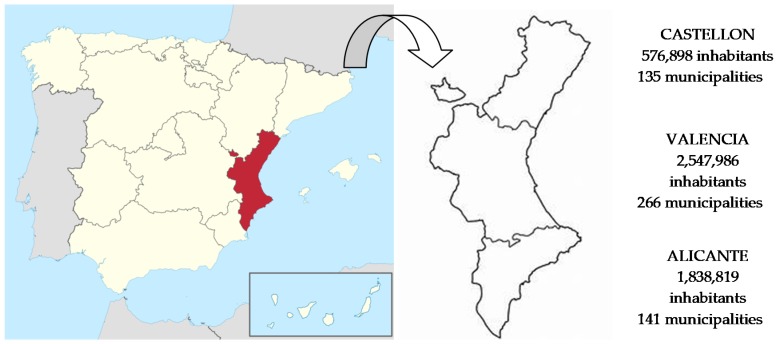
Valencia region. Location, population and number of municipalities. Source: Adapted by authors.

The Valencia region is located in the east and southeast of the Iberian Peninsula, with a surface area of 23,255 km^2^, and is approximately 4.6% of the surface area of Spain. It is composed of three provinces—Castellon, Valencia, and Alicante—and in 2018 had a population of 4,963,703 inhabitants (Figure 1), which represents 10.6% of the Spanish population [62]. The Valencia region has a government made up of various departments whose headquarters are in the city of Valencia, with offices in the cities of Alicante and Castellon. There are also three provincial councils, one in each province, whose mission is to provide infrastructure to the municipalities of their provinces, particularly those with smaller budgets. In the whole Valencia region, there are 542 municipalities.

## 3. Method

### 3.1. Measurement Technique: Content Analysis

Content analysis is the qualitative, objective, and systematic method [63,64] used in this study. Researchers analysed objective data from public tender documents obtained from calls for tenders, and quantified the results obtained by their transformation and parameterisation into indicators and metrics useful for interpretation of the results.

Many investigations on GPP have used this method. Some have focused on identifying environmental criteria in public procurement processes. Bouwer et al. (2006) [38] investigated the uptake of GPP practices in the EU 25 through the content analysis of a large sample. Kippo-Edlund et al. (2005) [65] studied the influence of environmental factors on tender award decisions in Sweden, Norway, Denmark, and Finland. Palmujoki et al. (2010) [66] analysed 156 tender documents to study environmental criteria in the acquisition of goods and services in Sweden and Finland during two periods (2005 and 2007).

In construction projects, Testa et al. (2016) [19] analysed content over a sample of 164 tenders collected from across Italy, to identify the degree of GPP in the construction sector in the country. Fuentes-Bargues et al. [20] studied the use of environmental criteria in Spanish public sector construction procurement over a sample of 100 cases of public procurement works for the years 2008–2011; and in 2018, studied the green public procurement of works tendered by Spanish universities in 2016 and 2017 [51].

Content analysis combined with other techniques was used by Faith-Ell (2005) [67] in the study of the application of environmental requirements in the procurement process of Swedish road maintenance, and by Adham and Siwar (2012) [34] in the study of green public purchasing in the Malaysian ICT sector.

### 3.2. Sample Selection

The Valencian region is one of the most important communities in terms of population, number of contracting authorities, and weight in the Spanish GDP. In this way, in-depth and meaningful data collection of GPP at the local and regional level is carried out.

**Figure 2:**
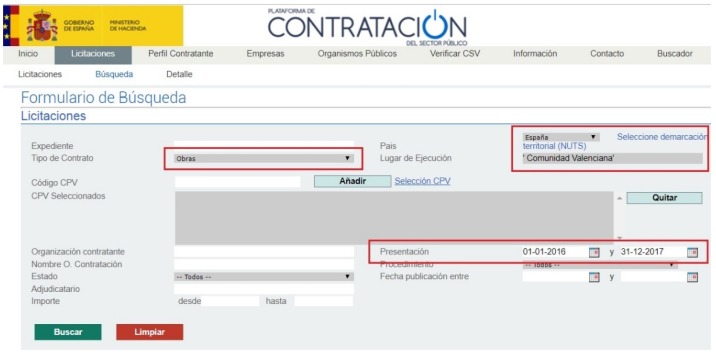
Spanish public sector procurement platform [68].

**Figure 3:**
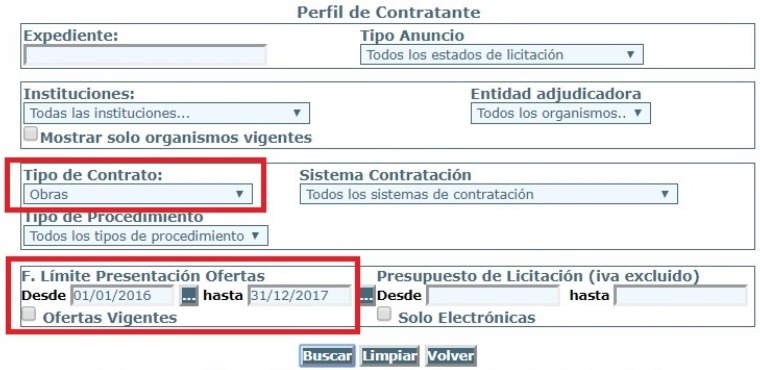
Valencia region public sector procurement platform [69].

The Spanish public administration has a web portal where it publishes a tender database. For this study, tenders from the Spanish public administration [68] (Figure 2), and from the Valencia regional public administration [69] (Figure 3) were selected. In some cases, details were also collected from the contracting authority websites (such as the city councils of Valencia and Castellon).

All available documentation was downloaded from the databases and websites, and tenders between 1 January 2016 and 31 December 2017 were analysed under the criteria of type of works.

Work tenders include, according to the definition of European and Spanish legislation, the construction, maintenance, and renovation of buildings, roads, airports, in all public facilities. Dates were chosen to include data for two full years of the economic crisis. The data collection process began in December 2017 and ended in July 2018.

The documents analysed were administrative specifications, tender notice, technical documents (with projects as complementary documentation). As with similar investigations, it was not possible to obtain all the documentation for the procedures [19,20,51].

The followed method was divided into six steps. The first step consisted of studying the project and the tendering documents. In step two, each sample case was analysed to locate any environmental criteria involved in the tenders. These environmental criteria were analysed and classified by subsector (civil engineering or building), contracting authority, geographical scope, and project budget. Thereafter, the weight of the environmental criteria was analysed and classified by subsector, geographical scope, and project budget. In the fourth step, the environmental criteria identified were related with other criteria used in the tendering process. Subsequently, a discussion and comparison with the results from other studies was included, and finally, the conclusions were presented.

### 3.3. Characteristics of the Sample

Some 1025 procedures were collected from work tender calls in the Valencia region in 2016 and 2017. After a revision of the documentation, only 967 were useful for the analysis (named N_T_ at Table 1), 675 from 2016, and 292 from2017. One hundred and forty–three contracting authorities were identified. Three hundred and forty–two procedures were tendered by administrations in the province of Valencia, 122 in Castellon, 262 in Alicante, and 241 from administrations covering the whole region.

The construction sector is divided into two subsectors. The building subsector includes all types of buildings: housing, factories, offices, schools, and sports facilities. The civil engineering subsector includes roads, ports, airports, railways, and water pipelines. In the sample, 395 projects belong to building subsector (named as N_B_) and 572 belong to the civil engineering subsector (named as N_C_).

The distribution for the contracting authorities with more than 10 procedures is presented in Table 1 and the list of all the contracting authorities is included in Appendix A.

Some 62.8% of the sample was composed of competitive tenders (EMAT)—and auctions formed the remaining 37.2%.

Five price intervals were established to analyse the influence of the project budget. Table 2 presents the number of projects included in each interval, both for each subsector and for the sample total.

Under Spanish public procurement regulations, there are several types of legal requirements that affect the time and complexity of the process, and a distinction is made between ordinary, urgent, and emergency processes. In the sample, 78.3% of the cases were processed under the ordinary procedure, whereas 21.5 % used the urgent procedure, and just 0.2% used the emergency procedure.

Another characteristic of the tendering process is the type of procedure. It is possible to distinguish between open procedures (all the firms reaching the requirements may participate) and negotiated procedures with, or without, advertising (only selected bidders can participate in such tenders, and the difference between ‘with’ and ‘without’ advertising is determined by the budget). For this study, 81.4% of the cases were tendered using open procedures, 4.9% with negotiated procedures, with advertising, and 13.7% by negotiated procedures without advertising.

The common procurement vocabulary (CPV) code is a system for the identification and categorisation of all economic activities that may be engaged in by means of public or competitive tender in the European Union [70]. This code enables classification of the scope of the project. Table 3 shows the main CPVs and how often they are used in the study sample.

## 4. Results

The results obtained in the analysis of the specifications indicate that 19.7% of the projects include references to environmental criteria. If we consider only the procedures tendered using several criteria (the most economically advantageous offer), the percentage rises to 31.3%. In the 190 procedures identified, it was found that contracting authorities use different definitions and descriptions to refer to very similar environmental criteria. Table 4 regroups the environmental criteria around the most commonly used definitions and descriptions (each public authority can choose and define the criteria used in the contracting process), identifying the number of times they have been used, both in the building and civil engineering subsectors.

The criterion most commonly used at a global level and for the building subsector is the ‘quality control’ criterion, used on 38 occasions, where both waste management and measures for environmental and landscape protection are analysed. For the civil engineering subsector, the most commonly used criterion is the ‘environmental action plan’, which is noteworthy since it has not been used with this description in any of the works of the building subsector.

Another noteworthy aspect is the criterion ‘energy efficiency and sustainability improvements’, which is the second most widely used environmental criterion in the building subsector, a criterion that focuses on the efficiency of the installed equipment (air conditioning, lighting, etc.).

The second environmental criterion in the global calculation, and the second in the civil engineering subsector, is ‘certified environmental accreditation’. Related to this, it is necessary to distinguish between procedures where the proof of possession of the certificate corresponding to the UNE/EN/ISO 14001 quality standard for environmental quality is simply requested (nine times) and others where certification is requested from an external organization that guarantees that an environmental management system is implemented on site.

Of the 190 procedures identified with environmental criteria, 88 belong to the building subsector (46.3%) and 102 projects to the civil engineering subsector (53.7%). With respect to the overall number of projects in each of the subsectors, civil engineering projects with environmental criteria represent 17.8%, whereas building projects with environmental criteria represent 22.3%.

Table 5 compares the number of dossiers with environmental criteria according to the province of action of the contracting authority and its percentage with respect to the overall number of dossiers. It can be observed how the provincial/local authorities inthe province of Valencia show the lowest implementation of GPP.

Table 6 shows the distribution of the procedures—including environmental criteria according to the geographical scope of the contracting authority and its percentage with respect to the total number of procedures for each subsector. It shows how regional contracting bodies use environmental criteria more (40.3% overall) than local and provincial authorities, and again how they are used slightly more often in the civil engineering subsector than in the building subsector.

Table 7 identifies the main contracting bodies that have used environmental criteria, the number of times, and the percentage with respect to the number of specifications of that contracting body in the study sample, both by subsector and globally.

The contracting authority with the largest number of files with environmental criteria is the ‘Valencia Regional Council for Housing, Public Works and Infrastructure’ (id. 103) with 38 projects, followed by the ‘Castellon City Council’ (id. 6) with 23, and the ‘Council for Education, Research, Culture and Sport’ (id. 98) and ‘Railways of the Generalitat Valenciana’ (id. 125) with 14.

In percentage terms referring to authorities with the largest number of projects in the study sample, those with the largestnumber of files with environmental criteria are: the ‘Castellon Port Authority’ with 100% (id. 3); the ‘University of Alicante’ with 92.9% (id. 139); the ‘Infrastructure Office of the Generalitat’ with 85.7% (id. 123); and the ‘Public Wastewater Sanitation Office’ (id. 124) and ‘Railways of the Generalitat Valenciana’ with 66.7%. With the exception of ‘Castellon City Council’ and the ‘University of Alicante’, these are independent administrations or, as in the case of the port authorities, under the Spanish Ministry of Public Works.

Table 8 shows the relationship between tenders with environmental criteria, the manner that bidders participate in the tender, and the type of administrative processing of the procedure. The results show that environmental criteria are hardly ever used in negotiated procedures and in urgent and emergency tenders.

If the analysis is performed from the point of view of the project budget as Table 9 shows, it can be affirmed that environmental criteria are more often used in both construction subsectors in projects with budgets between €1,000,001 and €10,000,000. It can be concluded that environmental criteria are included more often inthe building subsector than in the civil engineering subsector in projects over €200,000 and €1,000,000. For projects under €200,000, the use of environmental criteria is residual and the same in both subsectors.

According to the CPVs, there are 19 procedures with environmental criteria under the code 45,000,000 (which makes the 26.7% of the total procedures with this code), followed by code 45,233,252 with eight procedures (24.2%); and code 45,210,000 with seven procedures (20%). There is no relationship between specified CPVs and environmental criteria.

According to the description of the method of our study, in the fourth step, the weight of the environmental criteria was studied. The average weight of the environmental criteria is 4.12 points out of 100. The maximum weight of the environmental criteria was 26points out of 100 in a project where the environmental criteria was the substitution of current lighting with LED lighting (id. 53 Mislata City Council).

**Figure 4:**
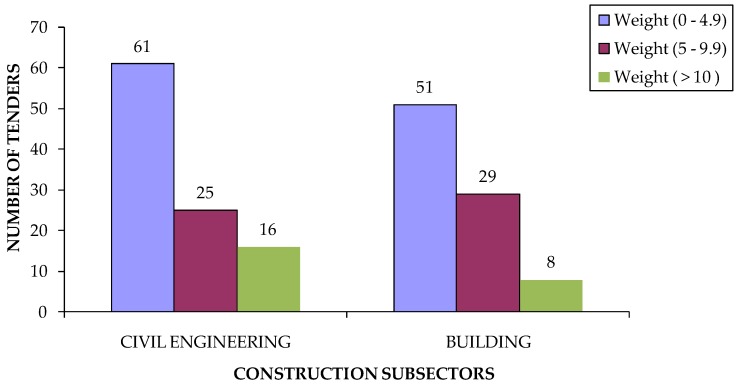
Number of tenders according to weighting of environmental criteria by construction subsector.

**Figure 5:**
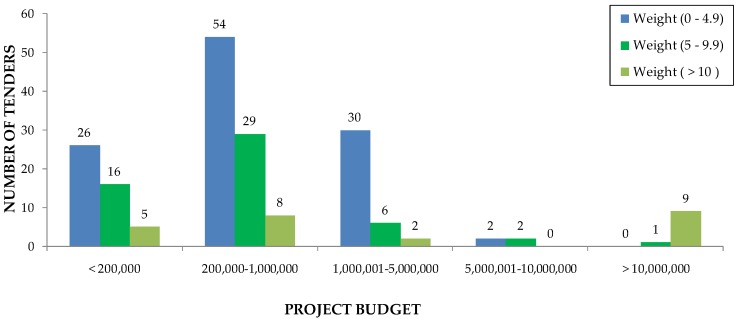
Number of tenders according to environmental criteria weighting by contract size (in euros).

Figure 4 shows that the weighting range for environmental criteria, for both subsectors, varies between 0 and 4.9 points in 100. If the comparison is made between the environmental criteria and the project budget (Figure 5), the results show that the most used weighting range for environmental criteria is between 0 and 4.9 points out of 100 for all the intervals of the budget, excluding projects with budgets greater than €10,000,000. As can be seen in Table 9, this type of project belongs to the civil engineering subsector.

As the fifth step of the method, the relationship between environmental criteria and other criteria of the tendering process is analysed. Table 10 presents the main criteria used and the average weight in the 190 projects identified with environmental criteria.

## 5. Discussion

Administrations in the Valencia region used environmental criteria when contracting 19.7% of the construction projects in 2016 and 2017. This percentage is similar to a recent study of construction projects for Spanish public universities for the same period (19.3%) [51]. These values are lower than the 40% average in the EU27 in 2012 as stated by Renda et al. [39], but similar to studies performed by Testa et al. in Italy [19] in 2010 (23%) and in 2012–2013 (19%) [44]. Some explanations have been made by other researchers [19,39,51,71], as there is a clear difference between Mediterranean countries and the ‘Green 7’ (Sweden, Germany, Austria, Denmark, United Kingdom, the Netherlands and Finland). Results obtained from the analysis of the call for tenders are more conservative than the results obtained by surveys of public administration managers.

Table 4 shows the description of the environmental criteria used in the contracts in the sample. All the criteria are assessed with value judgment criteria and the most often used criterion (38 times of 190) is ‘quality control’, which includes the control of waste and environmental and landscape protection measures.

Environmental plans (EPs) or environmental action plans (EAPs) continue as one of the main environmental criteria, in the same way as previous studies of GPP in Spain [2,51]. Some of the points included in the EP/EAPs are identification of work units that can generate impact during construction and operation; measures adopted to eliminate, reduce, and correct the impact; location of landfills; identification of applicable environmental legal requirements; and proposed systems of on-site environmental management. However, the standardization of this document is necessary because there is a variation between administrations.

In this studied sample, the possession of a certified environmental accreditation (mainly ISO 14001) has been used as environmental criteria, and so Valencian administrations must make an effort not to use it as an award criteria to accomplish the requirements of the European directives [22,23] and Spanish legislation [25,26,53].

If the environmental criteria identified are compared with the main criteria defined by the European Commission guide on GPP [55], it can be identified that the criterion ‘energy efficiency and sustainability improvements’ is quite often used (mainly in the building subsector). The use of criteria such as life cycle assessment (LCA) or carbon footprint (CF) is residual (specifically carbon footprint, which was used once). There are references or similarities to other criteria, such as the improvement criterion or the ‘use of materials and manufacturing techniques’, mainly in the civil engineering subsector.

The use of environmental criteria is slightly greater in the civil engineering subsector than the building subsector; and these results are aligned with previous research on GPP carried out in the Spanish construction sector [20,51] and in the Swedish construction sector [50].

Public administration influences the use of environmental criteria because, among other reasons, it has more resources and its technical staff are well trained [32,42,51,52,72,73]. The results of this research in the Valencia region lead to the same conclusions; the regional administrations use the most common environmental criteria more often than local and provincial administrations.

No conclusions on the use of environmental criteria can be associated with the geographical distribution of the administrations within the region. There are some administrations that use environmental criteria for all, or almost all, of their projects and others that never use them.

The CPV code, the method of participation, and the type of administrative processing have no influence on the use of environmental criteria in the contracting process. In fact, as previous studies [51] show, it can be concluded that environmental criteria are hardly ever used in negotiated procedures or in urgent and emergency processes.

As a consequence of the results and aligned to other studies on GPP both in Spain [20,51] and Europe [19,44,50,66], it can be affirmed that the use of environmental criteria in the tendering process is more common when the project budget is large and more accentuated in civil engineering projects. The use of environmental criteria in projects with budgets below €200,000 is almost residual (10.1%).

The average weighting of the environmental criteria in this study is 4.1 points out of 100, similar to the 3–5 points obtained by Palmujoki et al. [66] in their research in Sweden and Finland, as well as the 3.3 points obtained by Nissinen et al. [40] in Sweden, Finland, and Denmark. However, it is lower than in other studies, such as that conducted by Värnas et al. [50] for public works in Sweden, or by Igarashi et al. in Norway [43] for a sample of information and communication technology tenders, or a study made by Testa et al. in Italy [19] for a sample of public construction projects.

Recent studies on GPP in Spain reveal that the average weight of tenders from the Valencia region is lower than a sample of 100 public work projects throughout Spain (with a weight of 5.7 [20]), and also lower than the study of GPP for public works on Spanish universities (with an average weight of 6.5 out of 100 [51]). The obtained values enable affirming that the environmental criteria used in public procurement processes in the Valencia region show values similar to other studies in Spain and in nearby countries—but with significantly lower weights.

On the same trend, the importance of environmental criteria within the 190 projects of the sample with environmental criteria falls to eleventh place (after price, work program, description of construction process, enhancements, social criteria, guarantee period, health and safety procedures, and quality control), with a level of importance lower than previous studies [20,51,54].

Since there are not many studies at the local level on the state of GPP in public construction works, possible hypotheses that could justify this lower weight are the lack of knowledge on environmental matters of the technicians of the contracting authorities or the non-alignment of the guidelines of the different contracting authorities with European regulatory policies on sustainability. For this reason, future lines of research should be considered in two ways: on the one hand, to contrast the opinion and training of the technicians of the Valencian contracting authorities and, on the other hand, to carry out similar studies at a local level in Spain or elsewhere in order to compare the results.

As a result of the findings of the research and the discussion with previous works, some recommendations can be proposed to improve the current status. It is necessary to develop policies to regulate and encourage the use of environmental criteria in public works because there are considerable differences in practices in the wide spectrum of Valencian administrations. Among the actions to be taken, plans for the environmental training of technical staff, especially for local and provincial administrations, are necessary and urgent.

## 6. Conclusions

Valencian public administrations use environmental criteria in 19.7% of the works tendered with a low average weight within the contracting process (4.1 points out of 100). The main criteria used are ‘quality control’ (where control of waste and environmental and landscape protection measures are included) and ‘environmental action plan’. The use of environmental criteria in the Valencia regional administrations is higher in the civil engineering subsector, particularly in projects tendered by regional administrations. In projects with large budgets, both in the civil engineering and building subsector, the use of environmental criteria is similar.

This study analyses the level of GPP according to the documents of the public tendering process but has a limitation—it does not take into account the opinion of the technicians of the Valencian public authorities. Thus, a future line of research can be to analyse how the technicians of the Valencian public authorities select and subsequently assess environmental criteria. Also, other future research should apply the method to other public administrations, from Spain and/or other countries, to compare the current status of GPP and develop tools to improve green performance in the construction process for public works.

## Figures and Tables

**Table 1:** Contracting authorities of the study sample with more than 10 procedures.

ID	Contracting Authorities	N_B_	N_C_	N_T_
103	Valencia Regional Council for Housing, Public Works and Infrastructure	17	52	69
121	Alicante Provincial Council	6	51	57
12	Alicante City Council	10	36	46
96	Council for Agriculture, Environment, Climate Change and Rural Development	1	43	44
84	Valencia City Council	16	27	43
98	Council for Education, Research, Culture and Sport	36	0	36
6	Castellon City Council	12	21	33
120	Valencia Provincial Council	0	32	32
125	Railways of the Generalitat Valenciana	5	16	21
20	Benidorm City Council	6	15	21
74	San Vicente del Raspeig City Council	8	12	20
32	Elche City Council	7	12	19
47	Lliria City Council	8	11	19
57	Onteniente City Council	5	13	18
64	Picassent City Council	5	13	18
94	City of Arts and Sciences	17	0	17
61	Paterna City Council	8	7	15
5	Alzira City Council	7	7	14
139	University of Alicante	12	2	14
59	Oropesa del Mar City Council	5	8	13
42	Ibi City Council	5	6	11
27	Catarroja City Council	5	6	11
104	General University Hospital of Valencia	10	0	10
129	Valencian Institute for Social Action	10	0	10

N_T_ is the number total of procedures collected from the call for tenders with all the documentation for the analysis; N_B_ is the number of procedures from the building subsector, and N_C_ is the number of procedures from the civil engineering subsector.

**Table 2:** Distribution of the project budget of the sample of study for the building subsector, civil engineering subsector, and total.

Project Budget (€)	N_B_	N_C_	N_T_
<200,000	207	257	464
200,000–1,000,000	152	251	403
1,000,001–5,000,000	30	52	82
5,000,001–10,000,000	6	2	8
>10,000,001	0	10	10

**Table 3:** CPVs of the sample of study.

CPV	Frequency	Description
45,200,000	80	Works for complete or part construction and civil engineering work
45,000,000	71	Construction work
45,233,120	41	Road construction works
45,233,222	40	Paving works
45,210,000	35	Building construction work
45,233,252	33	Street paving works
45,233,140	31	Road works
45,220,000	25	Engineering works and construction works
45,215,200	21	Construction works of buildings for social services
45,212,200	21	Construction work for sports facilities
45,215,140	20	Hospitals
45,310,000	20	Electrical installation work

**Table 4:** Environmental criteria, number of times used, and its description in the study sample.

Description of the Environmental Criterion	Frequency that Environmental Criteria (EC_B_) is Mentioned in the Building Subsector	Frequency that Environmental Criteria (EC_C_) is Mentioned in the Civil Engineering Subsector	Total Frequency that Environmental Criteria (EC_T_) Is Mentioned	Description
Quality control	20	18	38	Control of waste management and environmental and landscape protection measures.
Certified environmental accreditation	9	22	31	Certificate issued by an accredited entity, guaranteeing the implementation of the management system on the work. Proof of possession of the certificate corresponding to the UNE/EN/ISO 14001 quality standard for environmental quality.
Environmental action plan	0	30	30	Identification of work units that can generate impact during construction and operation. Measures adopted to eliminate, reduce, and correct impact. Location of landfills. Identification of applicable environmental legal requirements. Proposed systems of good environmental management on site.
Programming and organisation of the works/study of the project	10	10	20	The accuracy in identifying and minimising the work units that can generate impacts, the proposal of work instructions that lead to improvements in the environment, and qualifying the physical organisation of work. The monitoring and control plan for the works must specify the methodology to be implemented for quality assurance. Improvements can refer to the execution during the construction process, or to the solutions or features that will be integrated into the final result and which will make the operation of the building more sustainable and environmentally friendly.
Energy efficiency and sustainability improvements	16	2	18	Improvements related to the energy and environmental efficiency of the equipment installed, and which result in a reduction in electrical consumption, and improve the performance of the new installation.
Waste management	13	2	15	Control of waste management.
Quality and environmental control	10	2	12	The contribution of an environmental management system will be assessed, including the technical and economic resources that the bidder intends to use for this purpose.
Use of materials and manufacturing techniques	0	9	9	The proposal for the use of materials and manufacturing techniques that achieve environmental improvements, such as the reuse of waste, recycling, reducing the emission of gases into the atmosphere, etc.
Environmental management Environmental criteria	5	3	8	Adequacy and development of the waste management plan. Environmental report. Registration of carbon footprint, offset, and CO_2_ absorption projects of the Spanish environment ministry. Measures that favour the reduction of waste by reusing it on site, including possible treatments for anew function.
Improvements in the project	2	4	6	These are not general measures. Environmental improvements specific to each project, such as: - Execution of works with open-air machinery with low noise emissions, - Reduction of greenhouse gas emissions with suppliers that generate these emissions close to the construction site, - Proof of the use of recycled stone material, Substitution of current lighting to Light Emitting Diode (LED)
Sustainability	3	0	3	The measures proposed by the company that involve improvements related to protecting the environment will be assessed. Improvements may be made during the construction process of the building (or to solutions or features that will be integrated into the final result) that will make the building more sustainable and environmentally friendly. The measures to be considered include those that improve the energy consumption of the building, those that lead to savings in water consumption, and the use of recycled materials or materials that are easily recoverable or reusable at the end of their useful life. It will also be favourably assessed if the timber, forest products, or processed products derived from wood, or other forest products that the company use in the works, have an International FSC (Forest Stewardship Council) or PEFC (Programme for the Endorsement of Forest Certification Schemes) certificate, or any other internationally recognised certificate confirming that the timber comes from sustainably managed forests.

**Table 5:** Environmental criteria by geographical scope of the contracting authority (GSVR) in the Valencia region.

Geographical Scope in the Valencia Region of the Contracting Authority (GSVR)	EC_T_	EC_T_/N_T_ (%)
Alicante province	36	13.7
Castellon province	34	27.9
Valencia province	23	6.7
Valencia region	97	40.3

**Table 6:** Environmental criteria by geographical scope of the contracting authority and by subsectors.

Geographical Scope of the Contracting Authority (GS)	Building Subsector			Civil Engineering Subsector			Total		
	EC_B_	EC_B_/N_B_ (%)	EC_B_/∑EC_T_ (%)	EC_C_	EC_C_/N_C_ (%)	EC_C_/∑EC_T_ (%)	EC_T_	EC_T_/N_T_ (%)	EC_T_/∑EC_T_ (%)
Local	35	15.5	42.7	47	13.0	57.3	82	14.0	43.2
Provincial	11	20.8	100	0	0.0	0.0	11	7.9	5.8
Regional	42	36.2	43.3	55	44	56.7	97	40.3	51.0

EC_B_ is the number of procedures with environmental criteria in the building subsector; EC_C_ is the number of procedures with environmental criteria in the civil engineering subsector; EC_T_ is the number of procedures with environmental criteria in the global construction sector; ∑EC_T_ is the total number of procedures with environmental criteria in the global construction sector (in this case 190 procedures); N_B_ is the number of procedures in the building subsector; N_C_ is the number of procedures in the civil engineering subsector; N_T_ is the number of procedures in the global construction sector.

**Table 7:** Distribution of environmental criteria by contracting authorities and by subsectors.

ID	Contracting Authorities	GS	EC_B_	EC_B_/N_B_ (%)	EC_C_	EC_C_/N_C_ (%)	EC_T_	EC_T_/N_T_ (%)
2	Alicante Port Authority	L	--	--	2	100	2	100
3	Castellon Port Authority	L	--	--	6	100	6	100
4	Valencia Port Authority	L	0	0	4	50	4	44.4
6	Castellon City Council	L	9	75	14	66.7	23	69.7
8	Alcoy City Council	L	--	--	2	40	2	40
12	Alicante City Council	L	1	10	5	13.9	6	13
18	Benicarlo City Council	L	0	0	1	25	1	20
21	Betxi City Council	L	2	100	0	0	2	50
30	Denia City Council	L	--	--	2	50	2	50
46	La Nucia City Council	L	--	--	1	100	1	100
53	Mislata City Council	L	--	--	1	100	1	100
57	Onteniente City Council	L	0	0	3	23.1	3	16.7
73	San Rafael del Rio City Council	L	1	100	--	--	1	100
74	San Vicente del Raspeig City Council	L	2	25	3	25	5	25
81	Torrente City Council	L	--	--	1	100	1	100
96	Council for Agriculture, Environment, Climate Change and Rural Development	R	0	0	11	25.6	11	25
97	Council for Social Welfare	R	2	22.2	--	--	2	22.2
98	Council for Education, Research, Culture and Sport	R	14	37.8	--	--	14	37.8
100	Council for Equality and Inclusive Policies	R	1	50	--	--	1	50
101	Council for Justice, Public Administration, Democratic Reforms and Public Freedoms	R	2	66.7	--	--	2	66.7
103	Valencia Regional Council for Housing, Public Works and Infrastructure	R	11	64.7	27	51.9	38	55.1
111	Alicante Health Department	L	1	25	--	--	1	25
113	Elda Health Department	L	3	75	--	--	3	75
118	Valencia Health Department. Arnau de Vilanova	L	2	66.7	--	--	2	66.7
119	Valencia Health Department. DrPeset	L	4	100	--	--	4	100
122	Castellon Provincial Council	P	1	25	--	--	1	25
123	Infrastructure Office of the Generalitat	R	6	85.7	--	--	6	85.7
124	Public Wastewater Sanitation Office	R	--	--	6	66.7	6	66.7
125	Railways of the Generalitat Valenciana	R	3	60	11	68.8	14	66.7
130	Valencian Institute of Modern Art	L	2	100	--	--	2	100
136	Presidency of the Generalitat Valenciana	R	0	0	--	--	0	0
137	Valencian Occupation and Training Service	R	1	33.3	--	--	1	33.3
139	University of Alicante	L	11	91.7	2	100	13	92.9
141	Polytechnic University of Valencia	L	4	100	0	0	4	80
142	University of Valencia	L	3	75	--	--	3	75
143	Valencian Waste Energy Use Agency	R	2	40	0	0	2	22.2

GS is geographical scope of the administration; L is local; P is provincial, and R is regional.

**Table 8:** Environmental criteria according to the type of administrative processing and participation approach.

**Type of Administrative Processing**	**Tenders with Environmental Criteria**	**Total Tenders**
Ordinary	150	757
Urgent	40	208
Emergency	0	2
**Method of Participation**	**Tenders with Environmental Criteria**	**Total Tenders**
Open	173	787
Negotiated with advertising	5	47
Negotiated without advertising	12	133

**Table 9:** Distribution of environmental criteria by project budget and by subsectors.

Project Budget (€)	EC_B_/N_B_ (%)	EC_C_/N_C_ (%)	EC_T_/N_T_ (%)
<200,000	10.1	10.1	10.1
200,000–1,000,000	30.3	17.9	22.6
1,000,001–5,000,000	60.0	38.5	46.3
5,000,001–10,000,000	50.0	50.0	50
>10,000,000	---	100	100

**Table 10:** Distribution of environmental criteria by project budget and by subsectors.

Criteria	Number of Times and Percentage	Criteria	Weight
Environmental criteria	190 (100%)	Price	55.1%
Price	190 (100%)	Work programme	20.3%
Work programme	124 (65.3%)	Description of the construction process	17.0%
Quality control	118 (62.1%)	Enhancements	16.2%
Analysis of the project	102 (53.7%)	Analysis of the project	11.4%
Health and safety procedures	73 (38.4%)	Social criteria	10%
Description of the construction process	67 (35.3%)	Enhancement of the guarantee period	8.4%
Enhancements	50 (26.3%)	Completion period	7.0%
Completion period	39 (20.5%)	Health and safety procedures	4.9%
Social criteria	22 (11.6%)	Quality control	4.5%
Enhancement of the guarantee period	20 (10.5%)	Environmental criteria	4.1%

## Abbreviations

CF	carbon footprint
CPV	common procurement vocabulary
DBFM	design-build-finance-maintain
EAP	environmental action plan
EPD	environmental product declaration
EMAT	economically most advantageous tenders
EC	European commission
EMS	environmental management systems
EP	environmental plan
EPD	environmental product declarations
EU	European union
FSC	forest stewardship council
GDP	gross domestic product
GPP	green public procurement
ICT	information and communication technology
LCA	life cycle assessment
LED	light emitting diode
OECD	organization for economic co-operation and development
PEFC	programme for the endorsement of forest certification schemes
SPP	sustainable public procurement
TOPSIS	technique for order of preference by similarity to ideal solution

## Appendix A

**ID**	**Contracting Authorities**	**N_B_**	**N_C_**	**N_T_**
1	Valencian Tourism Agency	2	0	2
2	Alicante Port Authority	0	2	2
3	Castellon Port Authority	0	6	6
4	Valencia Port Authority	1	8	9
5	Alzira City Council	7	7	14
6	Castellon City Council	12	21	33
7	Agullent City Council	0	3	3
8	Alcoy City Council	0	5	5
9	Alfafar City Council	0	1	1
10	Alfaz del Pi City Council	1	3	4
11	Algemesi City Council	1	1	2
12	Alicante City Council	10	36	46
13	Almassora City Council	2	4	6
14	Almussafes City Council	0	1	1
15	Aspe City Council	2	4	6
16	Bellreguard City Council	2	2	4
17	Benetusser City Council	0	1	1
18	Benicarlo City Council	1	4	5
19	Benicassim City Council	3	3	6
20	Benidorm City Council	6	15	21
21	Betxi City Council	2	2	4
22	Bocairent City Council	0	3	3
23	Bonrepos City Council	0	1	1
24	Burriana City Council	3	5	8
25	Camporrobles City Council	1	0	1
26	Carlet City Council	0	1	1
27	Catarroja City Council	5	6	11
28	Cocentaina City Council	3	5	8
29	Crevillente City Council	1	4	5
30	Denia City Council	0	4	4
31	El Puig de Santa Maria City Council	0	1	1
32	Elche City Council	7	12	19
33	Elda City Council	0	1	1
34	Enguera City Council	1	0	1
35	Estivella City Council	1	0	1
36	Favara City Council	1	1	2
37	Finestrat City Council	0	1	1
38	Gandia City Council	1	5	6
39	Gestalgar City Council	0	1	1
40	Godella City Council	0	1	1
41	Guardamar del Segura City Council	0	2	2
42	Ibi City Council	5	6	11
43	L’Eliana City Council	0	3	3
44	L’Alcora City Council	0	1	1
45	L’Olleria City Council	0	2	2
46	LaNucia City Council	0	1	1
47	Lliria City Council	8	11	19
48	Manises City Council	3	1	4
49	Manuel City Council	0	1	1
50	Massamagrell City Council	0	1	1
51	Massanassa City Council	1	1	2
52	Meliana City Council	2	1	3
53	Mislata City Council	0	1	1
54	Moncofa City Council	0	1	1
55	Mutxamel City Council	1	2	3
56	Novelda City Council	0	1	1
57	Onteniente City Council	5	13	18
58	Orihuela City Council	1	2	3
59	Oropesa del Mar City Council	5	8	13
60	Paiporta City Council	0	5	5
61	Paterna City Council	8	7	15
62	Peñiscola City Council	3	6	9
63	Petrer City Council	0	1	1
64	Picassent City Council	5	13	18
65	Puzol City Council	0	3	3
66	Quart de Poblet City Council	1	1	2
67	Requena City Council	1	2	3
68	Ribarroja del Turia City Council	1	0	1
69	Rojales City Council	2	1	3
70	Sagunto City Council	2	6	8
71	San Antonio de Benageber City Council	0	1	1
72	San Joan Moro City Council	1	0	1
73	San Rafael del Rio City Council	1	0	1
74	San Vicente del Raspeig City Council	8	12	20
75	Sedavi City Council	0	2	2
76	Segorbe City Council	2	1	3
77	Silla City Council	2	0	2
78	Sollana City Council	0	1	1
79	Sueca City Council	1	0	1
80	Torreblanca City Council	1	3	4
81	Torrente City Council	0	1	1
82	Torrevieja City Council	2	2	4
83	Utiel City Council	1	1	2
84	Valencia City Council	16	27	43
85	Valles City Council	2	0	2
86	Vilafames City Council	0	4	4
87	Villanueva de Castellon City Council	1	1	2
88	Villarreal City Council	2	2	4
89	Vinaros City Council	2	4	6
90	Xalo City Council	1	0	1
91	Xativa City Council	3	0	3
92	Xirivella City Council	2	0	2
93	Xixona City Council	0	1	1
94	City of Arts and Sciences	17	0	17
95	Agrarian Council of Sagunto	0	1	1
96	Council for Agriculture, Environment, Climate Change and Rural Development	1	43	44
97	Council for Social Welfare	9	0	9
98	Council for Education, Research, Culture and Sport	37	0	37
99	Council of Finance and Economic Model	4	0	4
100	Council for Equality and Inclusive Policies	2	0	2
101	Administration, Democratic Reforms and Public Freedoms	3	0	3
102	Council of Universal Health and Public Health	4	0	4
103	Valencia Regional Council for Housing, Public Works and Infrastructure	17	52	69
104	General University Hospital of Valencia	10	0	10
105	Provincial Hospital Consortium Hospital of Castellon	2	0	2
106	Marina Baja Water Supply and Sanitation Company	0	3	3
107	Valencian provincial Company for the Fire and Rescue, Prevention and Extinction Service	4	0	4
108	Castellon Provincial Firefighters	1	0	1
109	Valencia 2007 Company	1	3	4
110	Valencian Media Corporation	2	0	2
111	Alicante Health Department	4	0	4
112	Elche Health Department	1	0	1
113	Elda Health Department	4	0	4
114	Requena Health Department	1	0	1
115	Sagunto Health Department	3	0	3
116	Valencia Health Department Clinico	4	0	4
117	Valencia Health Department La Fe	2	0	2
118	Valencia Health Department Arnau de Vilanova	3	0	3
119	Valencia Health Department Dr. Peset	4	0	4
120	Valencia Provincial Council	0	32	32
121	Alicante Provincial Council	6	51	57
122	Castellon Provincial Council	4	0	4
123	Infrastructure Office of the Generalitat	7	0	7
124	Public Wastewater Sanitation Office	0	9	9
125	Railways of the Generalitat Valenciana	5	16	21
126	Foundation of the Valencian Community MARQ	3	0	3
127	Municipal Sports Foundation of Valencia city	0	3	3
128	Dr. Moliner Hospital	2	0	2
129	Valencian Institute of Social Action	10	0	10
130	Valencian Institute of Modern Art	2	0	2
131	Valencian Institute of Business Competitiveness	0	1	1
132	Central Supply Markets of Valencia	1	0	1
133	Valencia Music, Congress and Orchestra Palace	1	0	1
134	Arts Palace “Reina Sofía”	1	0	1
135	Singular Parks and Gardens and Municipal School of Gardening and Landscape of the Valencia city	1	2	3
136	Presidency of the Generalitat Valenciana	1	0	1
137	Valencian Occupation and Training Service	3	0	3
138	Department of Accounts of the Generalitat	1	0	1
139	University of Alicante	12	2	14
140	University Miguel Hernandez (Elche)	4	1	5
141	Polytechnic University of Valencia	4	1	5
142	University of Valencia	4	0	4
143	Valencian Waste Energy Use Agency	5	4	9
	TOTAL	395	572	967
